# Detection and Full-Length Genome Characterization of Novel Canine Vesiviruses

**DOI:** 10.3201/eid2108.140900

**Published:** 2015-08

**Authors:** Vito Martella, Pierfrancesco Pinto, Eleonora Lorusso, Barbara Di Martino, Qiuhong Wang, Vittorio Larocca, Alessandra Cavalli, Michele Camero, Nicola Decaro, Krisztián Bányai, Linda J. Saif, Canio Buonavoglia

**Affiliations:** Università Aldo Moro di Bari, Valenzano, Italy (V. Martella, P. Pinto, E. Lorusso, V. Larocca, A. Cavalli, M. Camero, N. Decaro, C. Buonavoglia);; Università degli Studi di Teramo, Teramo, Italy (B. Di Martino);; The Ohio State University, Wooster, Ohio, USA (Q. Wang, L.J. Saif);; Hungarian Academy of Sciences, Budapest, Hungary (K. Bányai)

**Keywords:** calicivirus, vesivirus, dogs, genome, canine, detection, characterization, novel, viruses, Italy

## Abstract

Vesiviruses have been detected in several animal species and as accidental contaminants of cells. We detected vesiviruses in asymptomatic kennel dogs (64.8%) and symptomatic (1.1%) and asymptomatic (3.5%) household dogs in Italy. The full-length genome of 1 strain, Bari/212/07/ITA, shared 89%–90% nt identity with vesiviruses previously detected in contaminated cells.

Members of the family *Caliciviridae* are small (30–35 nm in diameter), nonenveloped viruses with a single-stranded, positive-polarity RNA genome of 7.4–8.3 kb. The family consists of the genera *Vesivirus*, *Lagovirus*, *Norovirus*, *Sapovirus*, and *Nebovirus* as well as unassigned caliciviruses (*1*,*2*).

Vesiviruses were originally identified in 1932 in California, USA, in domestic swine with vesicular disease. Since then, vesiviruses have been described in several animal species and humans, and they have been associated with a variety of clinical signs and lesions, including abortion, hepatitis, respiratory disease, diarrhea, myocarditis, encephalitis, mucosal ulcerations, vesicular lesions, and hemorrhagic syndromes (*3*–*5*). Unlike other caliciviruses, vesiviruses appear to readily cross host species barriers, and the marine ecosystem is believed to constitute a large reservoir of vesiviruses for terrestrial animals (*5*).

Vesiviruses have occasionally been detected in dogs with diarrhea and, in some instances, in dogs with glossitis, balanitis, or vesicular vaginitis (*6*–*9*). However, with the exception of a canine calicivirus, strain 48 (*9*), the caliciviruses detected in dogs have been feline viruses (*6*,*7*). The prototype canine calicivirus (CaCV) strain 48 was identified in Japan in 1990; the virus was isolated from a 2-month-old pup with intermittent watery diarrhea (*9*). Strain 48, which is antigenically and genetically unrelated to feline caliciviruses, was tentatively proposed as a true CaCV in the *Vesivirus* genus (*10*). Antibodies to strain 48 have been detected in 57.0% of dogs in Japan (*11*) and 36.5% of dogs in South Korea (*12*), but no information is available regarding the circulation of analogous viruses in dogs elsewhere.

In 2003, a novel vesivirus (strain 2117) genetically similar to CaCV strain 48 was accidentally isolated as a contaminant in Chinese hamster ovary (CHO) cell cultures by a pharmaceutical company in Germany (*13*). These cells are mostly used by biotech companies for the production of recombinant drugs; possible sources of contamination included reagents used for cell cultivation, such as porcine-derived trypsin or fetal bovine serum.

The limited information available does not clarify whether vesiviruses play a role as enteric pathogens in dogs. Considering the ability of vesiviruses to cross the host species barriers (*5*) and the close social interactions between humans and dogs, it is essential to determine whether dogs harbor viruses with a zoonotic potential. To further investigate the molecular epidemiology of vesiviruses, we screened fecal specimens from asymptomatic dogs and from dogs with diarrhea in Italy.

## The Study

In 2007, we collected 385 samples from dogs in Bari, Italy. A total of 183 samples were fecal specimens from household dogs (1–6 months of age) hospitalized with signs of mild to severe gastroenteritis (collection A); 88 were rectal swab specimens from clinically healthy juvenile and adult dogs housed in 4 separate shelters (collection B); and 114 were fecal swab specimens from asymptomatic household dogs (1–6 months of age) receiving routine care at 2 veterinary clinics (collection C).

By using reverse transcription PCR with the broadly reactive consensus primers for caliciviruses, p289/p290 (*14*), and strain 48–specific primers 493F–526R (Table), we detected vesivirus RNA in 1.1% (2/183), 64.8% (57/88), and 3.5% (4/114) of collection A, B, and C samples, respectively. Partial RNA-dependent RNA polymerase sequences, obtained by using the primers p289/p290 (*14*), were determined for 10 samples. The sequences shared closest nucleotide identity (90.7%–92.6%) with vesivirus strains 2117, Geel/2008, Allston/2008/USA, and Allston/2009, which were identified as contaminants in CHO cells; the sequences shared 73.6%–74.8% nt identity with CaCV strain 48. We determined the full-length genomic sequence (8,453 nt) of 1 of the canine vesivirus strains, Bari/212/07/ITA, by using consensus primers and 3′ and 5′ RACE protocols (Invitrogen Ltd, Milan, Italy); the sequence was deposited in GenBank (accession no. JN204722). Primers used for virus detection and sequencing are listed in the Table.

**Table:** Primers used in study of the detection and full-length genome characterization of novel canine vesiviruses

Primer	Position*	Sequence	Sense	Use†
493F	5127–5150	GGT TTG CCA TCT GGC ATG CCG CTA	+	Detection
526R	5793–5816	AGC CAT VGC TCA RTT CTC AAA CAC	−	Detection
p289	5271–5292	TGA CAA TGT AAT CAT CAC CAT A	−	Detection
p290	4962–4984	GAT TAC TCC AAG TGG GAC TCC AC	+	Detection
501F	554–577	GTC TTG TGC TCT VTA CGA CAM ATG	+	Sequencing
VN3T20	3′- poly A	GAG TGA CCG CGG CCG CT_20_.	−	3′ RACE
502F	1215–1234	ATG ATW ATT GAH AAC CAY GA	+	Sequencing
509F	2904–2923	TAC GAT ATG GCY TGG GCY CT	+	Sequencing
510F	3411–3432	GAT GAT GAG TAC GAT GAR TGG A	+	Sequencing
511F	3588–3607	GAA GAC GTC ACC RTA ATT GG	+	Sequencing
513F	3876–3898	GTT ACG TTC RAT GGY GAA TTG GC	+	Sequencing
515F	4314–4338	CAC GTG TCA CCA GCA CAC RTD GAT G	+	Sequencing
504R	1599–1618	ACC ACG CTY TCR TTS GAC CA	−	Sequencing
528R	2887–2909	CTT GTC ATC TTA GTG TAC AAT GA	−	Sequencing
506R	1680–1702	AG GTT GGT RAC NGC RTC AAT GTC	−	Sequencing
508R	1974–1993	GT GTA GGC RYC GTG GTG GTC	−	Sequencing
512R	3588–3608	GCC AAT TAY GGT GAC RTC WTC	−	Sequencing
536F	714–732	TAC GAT CTT GCA ATC AAT G	+	Sequencing
GE72F	1915–1935	CCT ATG CCA TTG CGT CTA GAC	+	Sequencing
GE73R	2951–2970	CAG CCT TAA GTG CCT GCC AC	−	Sequencing
SEQ100	5634–5653	TGT CGC CAA ATG TTG ATG AG	+	Sequencing
SEQ101	6343–6360	TTG CCA CAG GCA CTC AGC	+	Sequencing
SEQ102	6860–6877	GGA AAC ACG TGG TGG TCA	+	Sequencing
SEQ103	7487–7504	AAG TAG AAT GAT TGG TGA	+	Sequencing
SEQ104	8063–8082	GAG TTT GAC AAG ATG AAC AG	+	Sequencing
GSP1	497–520	GCT TCA GAG ATC AGA ATA TCG TTG	−	5′ RACE
GSP2	366–385	GTG GTC AGA GCC TTG GTC AG	−	5′ RACE

*Position is based on the sequence of strain Bari/212/07/ITA (GenBank accession no. JN204722). †RACE, rapid amplification of cDNA ends.

**Figure 1:**
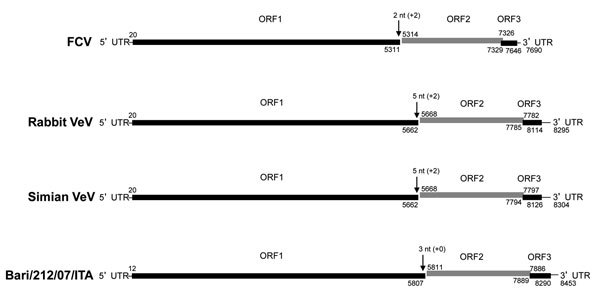
Genome organization of vesiviruses (VeVs). The genomic organization and open reading frame (ORF) usage are shown for representative viruses in the main VeV genetic groups: feline calicivirus (FCV) strain F9 (GenBank accession no. M86379), rabbit VeV (GenBank accession no. AJ866991), simian VeV strain Pan1 (GenBank accession no. AF091736), and canine VeV Bari/212/07/ITA. Numbers above and below the genome bar indicate the nucleotide (nt) position of the ORF initiation and termination, respectively. UTR, untranslated region.

**Figure 2:**
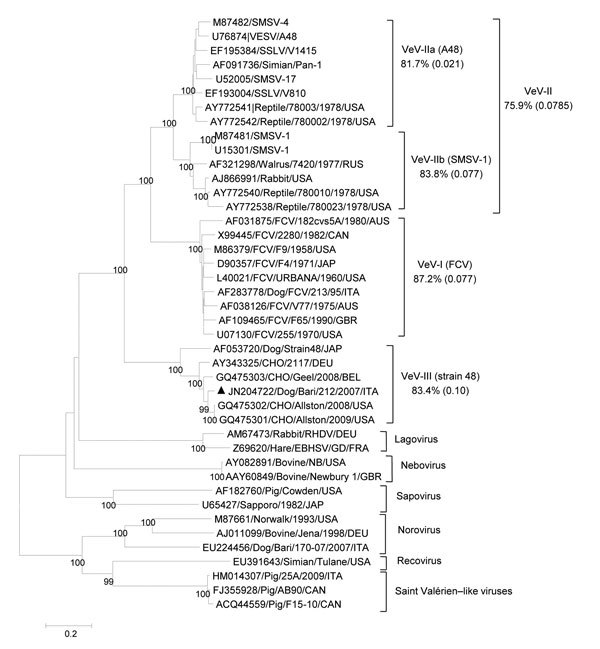
Phylogenetic tree based on the complete amino acid sequence of the capsid protein of vesiviruses (VeVs). The tree was constructed by using a selection of feline calicivirus strains and all of the VeV strains available in the GenBank database. In addition, viruses representative of the other established and candidate calicivirus genera were included. VeV groups were defined on the basis of distance matrix comparison and phylogenetic clustering. The mean identity among strains of the main genetic groups (indicated by Roman numerals and a letter or both) is shown. Numbers in parentheses indicate SDs. Black triangle indicates the canine VeV strain Bari/212/07/ITA. DEU, Germany; EBHSV, European brown hare syndrome virus; SMSV, San Miguel sea lion virus; SSLV, stellar sea lion virus; RHDV, rabbit hemorrhagic disease virus; VESV, vesicular exanthema of swine virus. Scale bar represents the number of amino acid substitutions per site.

The full-length genomic sequence of strain Bari/212/07/ITA shared 89%–90% nt identity with sequences of viruses identified as CHO cell contaminants; it shared only 71.0% nt identity with the prototype CaCV strain 48. Three open reading frames (ORFs) were predicted by sequence analysis of the nucleotide sequence and by comparing results with the genomic organization and ORFs of other vesiviruses (Figure 1). ORF1 was 5,796 nt in length (nt 12–5807) and encoded a 1,921-aa polyprotein. The ORF1 stop codon was followed by 3 nt and then by the ORF2 start codon. ORF2 was 2,079 nt in length (nt 5811–7889) and encoded a 692-aa capsid protein. ORF3 was 405 nt in length (nt 7886–8290) and encoded a 134-aa protein. ORF2 overlapped with ORF3 by 4 nt (Figure 1). The 163-nt 3′ untranslated region of CHO cell–associated strain 2117 was shorter than the 235-nt region in strain 48. The full-length ORF1-encoded polyprotein of Bari/212/07/ITA shared highest amino acid identity (98.5%–98.9%) with CHO cell–associated vesiviruses; it shared 80.5% aa identity with the ORF1-encoded polyprotein of canine strain 48 and <56% aa identity with other vesiviruses. Within the ORF2-encoded protein, the capsid cleavage motive FRAES (aa 155–159) was conserved. As observed, in CHO cell–associated strain 2117 and canine strain 48, a 7-aa insertion (KTIKSQV) was present in the conserved region D. The full-length capsid protein of Bari/212/07/ITA shared 92.6%–92.9% aa identity with the CHO cell–associated vesivirus strains Allston/2009/USA and Allston/2008/USA from the United States, 90.0% aa identity with the CHO cell–associated isolate Geel/2008 from Belgium, 86.3% aa identity with the CHO cell–associated isolate 2117 from Germany (*13*), and only 70.3% aa identity with the prototype CaCV strain 48 isolated in Japan in 1990 (*9*). Strain Bari/2012/07/ITA shared <35.8% aa identity with other vesiviruses (Figure 2).

In previous investigations in dogs, strain 48–like vesiviruses were detected in only 2 (1.7%) of 119 samples: a fecal specimen from a dog with signs of enteric disease and a tonsillar swab specimen from a dog with respiratory disease (*11*). However, serologic investigations suggested that vesiviruses actively circulate in the canine population (*11*,*12*). In our virologic investigations, vesivirus RNA was detected in 64.8% (57/88) of dogs housed in 4 shelters but in only 1.1% (2/183) and 3.5% (4/114) of symptomatic and asymptomatic household dogs, respectively. These findings demonstrate that canine vesiviruses can be widespread in some settings or populations (e.g., kennels or shelters) where high population densities create favorable epidemiologic situations for circulation of some microorganisms.

Of interest, partial sequence analysis of the RNA-dependent RNA polymerase fragment of several of the dog-derived strains and full-length genomic sequencing of strain Bari/212/07/ITA showed that these canine vesiviruses were more similar to some vesiviruses found as contaminants of CHO cell cultures than to the prototype CaCV strain 48. Contamination of CHO cells by vesiviruses was documented in 2003 in Germany (*13*), 2008 and 2009 in the United States, and 2009 in Belgium. The 2008–2009 contamination in the United States was estimated to cost US $100–300 million in lost revenues to the biotech company because production was interrupted to provide adequate sanitation and maintenance of the bioreactors (*15*). In addition, these contaminations have raised concerns for the potential exposure of humans to these novel viruses because vesiviruses can readily cross the host species barrier (*5*).

## Conclusion

Our findings show that genetically heterogeneous vesiviruses are common in dogs in Italy. Further studies are necessary to understand the ecology of this group of vesiviruses; that is, it must be determined whether vesiviruses circulate in humans or other animal species and, above all, whether they are associated with disease in humans, animals, or both.
